# Handgrip Strength and All-Cause Mortality in Middle-Aged and Older Koreans

**DOI:** 10.3390/ijerph16050740

**Published:** 2019-03-01

**Authors:** Eun-Jung Bae, Na-Jin Park, Hae-Sook Sohn, Yun-Hee Kim

**Affiliations:** 1 Division of Nursing, Dongnam Institute of Radiological & Medical Sciences, Busan 46033, Korea; beaulife80@gmail.com; 2 Department of Nursing, Pukyong National University, Busan 48513, Korea; 3 School of Nursing, University of Pittsburgh, Pittsburgh, PA 15261, USA; parknj@pitt.edu; 4 Department of Preventive Medicine, Inje University, Busan 47392, Korea; pmshs@inje.ac.kr

**Keywords:** handgrip strength, mortality, Korean

## Abstract

Aging-related decline in handgrip strength has been associated with adverse functional and metabolic morbidity and mortality. Korea is one of the fastest aging countries, and the prospective relationship of handgrip strength with all-cause mortality in Korean adults has not been studied. We conducted a prospective observation study to examine whether baseline handgrip strength predicted mortality over eight years of follow-ups in Korean adults aged 45 years or older. We analyzed the nationwide survey data based on 9393 Korean adults (mean age of 61 ± 10.7 years) from the 2006–2014 Korean Longitudinal Study of Aging. The mean handgrip strength values measured using a dynamometer, and were divided into quartiles for each gender. Cox models were conducted in order to estimate the hazard ratios (HRs) of all-cause mortality with 95% confidence intervals (CIs) in relation to handgrip strength adjusting for covariates. There was a robust independent relationship between a weaker handgrip strength and higher all-cause mortality in both women and men, adjusting for selected covariates (e.g., age, income, smoking, exercise, and comorbidities). Compared to the strongest quartile (i.e., reference), women and men in the weakest group had higher HRs of mortality, 2.5 (95% CI: 1.7–3.8) vs. 2.6 (95% CI: 1.8–3.9), respectively. The robust independent relationships between weaker handgrip strength and higher all-cause mortality found in the study suggest that simply assessing and monitoring the handgrip strength during adulthood demonstrates great potentials for the public health of aging populations, and protects against premature death in Korean adults.

## 1. Introduction

One of the prominent features of aging is the changes in body composition, with reduced lean body mass and increased fat mass. Skeletal muscle is one of the major components of lean body mass, and a loss of skeletal muscle mass and strength (i.e., sarcopenia and dynapenia) progressively occurs with aging [1]. Lower levels of skeletal muscle mass and strength have shown strong associations with increased risks of morbidity and mortality in older adults [2,3]. Changes in skeletal muscle mass and strength have clinically-meaningful functional and metabolic consequences, such as frailty, disability, cardiovascular disease, and diabetes [4,5,6]. To date, there remains debate about how to measure skeletal muscle mass and strength, which are important for healthy aging [7].

Handgrip strength is quick and easy to measure, and is inexpensive. It is therefore attractive as a tool to stratify the risk of developing cardiovascular disease or the possibility of death from an incident illness [8]. Handgrip strength is a measure of the maximum static force that a hand can squeeze using a dynamometer [9]. Methods for evaluating handgrip strength have varied across previous studies. The maximal value of one-hand measurements or the mean value of both hands are often used in handgrip strength assessments [8,10,11]. Furthermore, the handgrip strength value obtained may differ among evaluation methods and tools [9]. Growing evidence suggests that changes in skeletal muscle strength assessed by handgrip strength may represent age-related changes in biological vitality and physical function [10,11]. Longitudinal studies suggest that poor handgrip strength is a powerful predictor of the increased risks of future disability, morbidity, and mortality [12,13]. Greater levels of handgrip strength have been associated with lower risks of cardiovascular disease, all-cause and cardiovascular mortality, physical function, and frailty [3,5,10]. In a previous study of Korean adults, it was also reported that the increased strength of the handgrip is associated with a lower degree of cardiovascular risk [14]. This relationship between handgrip strength and future mortality has been found not only in older adults [6], but also in middle-aged and young adults [15], indicating its long-term health implications throughout a lifespan.

Skeletal muscle strength and handgrip strength are affected by multiple factors, such as demographics (e.g., age and gender), socioeconomic variables (e.g., income and employment), lifestyle and health behaviors, and health status/comorbidities [13,16,17]. Previous literature clearly demonstrates the distinct differences in handgrip strength by age, gender, and ethnicities or nationalities [3,8,17,18]. A weak handgrip strength was defined as <26 kg for men and <18 kg for women, or as the lower 20th percentile for handgrip strength of the study population by the Asian Working Group of Sarcopenia (AWGS) [19]. European Working Group on Sarcopenia in Older People 2 (EWGSOP2) defined <27 kg for men and <16 kg for women as weak handgrip strength [20]. The cut-off values for weak handgrip strength in Japan were <30.3 kg for men and <19.3 kg for women, which represented <25% of the participants [21], and in Taiwan, they were <22.4 kg for men and <14.3 kg for women [22]. There are a few studies that have assessed normative handgrip strength in Koreans [8,23]. Kim et al. [8] proposed the cut-off value for weak handgrip strength as <28.9 kg for men and <16.3 kg for women, according to the EWGSOP definition. In addition, Yoo et al. [23] reported that the cut-off values of weak handgrip strength in elderly healthy populations were 28.6 kg and 16.4 kg for Korean men and women, respectively. As the definition of low handgrip strength varies, a low handgrip strength prevalence has also been presented to vary from 13.5% to 39.9% [24]. The prevalence of low handgrip strength in healthy Korean elderly women was reported to be 30.1% [25].

Korea is one of the fastest aging developed countries, and previous studies focusing on older adults in Korea have demonstrated significant relationships of handgrip strength with metabolic syndrome, osteoporosis, fracture, cognitive impairment, and depression [11,26,27,28]. However, little is known about the prospective relationship between handgrip strength and all-cause mortality in middle aged and older Korean adults. Using a population-based national sample of Korean adults, the purpose of this study was to examine whether the baseline handgrip strength would be associated with all-cause mortality over an eight-year follow-up after controlling for selected covariates.

## 2. Materials and Methods

### 2.1. Study Design and Population

**Figure 1:**
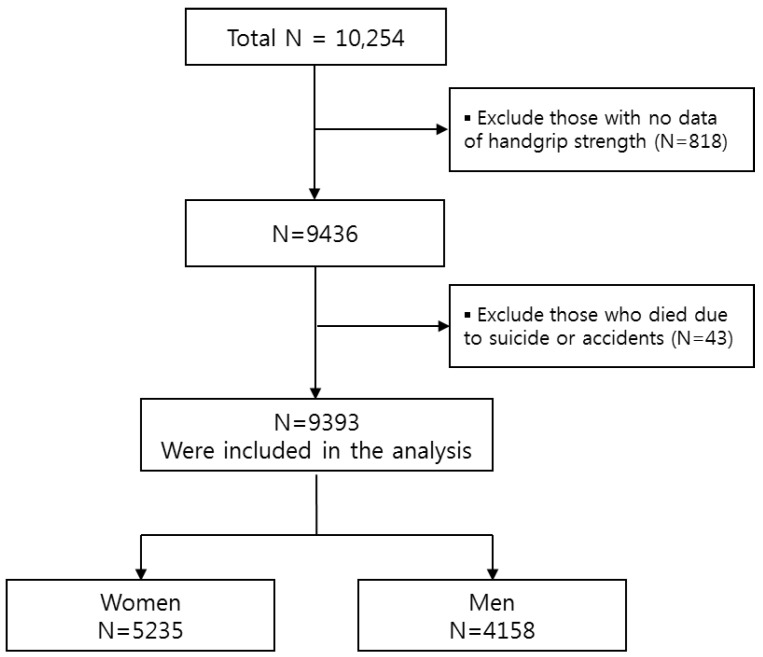
The flow diagram of participant inclusion and exclusion.

This prospective observation study is a secondary analysis of population-based survey data from the 2006–2014 Korean Longitudinal Study of Aging (KLoSA). KLoSA is a panel study involving 10,254 adults aged 45 years or older in 2006, in order to address sociodemographic factors, economic activities, health behaviors, health status/comorbidities, and other variables related to aging and health. The Korea Employment Information Service conducted the KLoSA survey using a multi-stage, stratified sampling based on the geographical areas and housing types across the nation. Trained interviewers visited participants’ homes and collected data through computer-assisted face-to-face interviews. All of the participants provided written informed consent, and the survey protocol was approved by the Institutional Review Board of the Statistic Korea (approval number: 336052). For the study, we included 9436 participants who had data for handgrip strength. We further excluded 43 deaths due to suicide or accidents, so as to focus on natural aging and death, resulting in a total sample size of 9393 for the primary analyses (Figure 1).

### 2.2. Measures

All-cause mortality was the outcome of the study, and was identified by follow-up surveys conducted every two years, up until 2014. Death over a maximum follow-up period of eight years was confirmed by family interviews and death certificates. Data on the specific causes of death were not available in the KLoSA dataset.

The handgrip strength was measured twice for each hand alternatively, using a dynamometer (Hand Grip Meter 6103, Tanita, Tokyo, Japan) to the nearest 0.1 kg. Participants were asked to sit or stand up, with their elbows by their side fixed at a 90-degree angle with their wrist in a neutral position, and then were asked to squeeze the dynamometer with each hand as hard as possible for five seconds. For the primary analyses, we used the average of the maximum values from both the left and the right hand of each participant. The average values of handgrip strength were divided into quartiles by each gender (women: <16.8, 16.8–19.9, 20.0–22.9, and ≥23.0 kg; and men: <28.5, 28.5–32.7, 32.8–37.2, and ≥37.3 kg) [8,11,14]. Subsequently, the same quartile groups of women and men were combined as gender-specific quartiles of handgrip strength.

The potential covariates that were selected were self-reported baseline characteristics of socio-demographics, health behaviors, and comorbidities. The baseline age was categorized into three groups, 45–54, 55–64, and ≥65 years old. Household income was divided into quartiles, then categorized to three groups (i.e., quartile 1 as low, quartiles 2 and 3 as middle, and quartile 4 as high). Participants were also asked to report their education level, marital status, residential area, and employment. Responding to one simple question for each selected health behavior, participants reported smoking and drinking (never, former, or current), regular exercise and eating breakfast (yes or no), height, and weight. The body mass index (BMI) at baseline was computed by weight (in kilograms)/height^2^ (in meters) and categorized into four groups, namely: underweight (<18.5 kg/m^2^), normal (18.5–24.9 kg/m^2^), overweight (25–29.9 kg/m^2^), and obese (≥30 kg/m^2^). Health status/comorbidities at baseline included hypertension, diabetes, cardiac disease, cerebrovascular disease, cancer, and depression, the total number of which was also presented as 0, 1, 2, or ≥3.

### 2.3. Statistical Analyses

Multiple imputation was used to handle the missing values of the major study variables identified (<20%) prior to the primary analyses. The number of imputations was determined to be five. The baseline characteristics were presented as frequency, percentage, and 95% confidence intervals (CIs) for the categorical variables, and as mean ± standard deviation and 95% CIs for the continuous variables. Distributions of the study participants by baseline characteristics were compared by using Chi-square tests and one-way analysis of variance (ANOVA) for the categorical and continuous variables, respectively. The mortality rates per 1000 person–years and 95% CIs were calculated by handgrip strength quartiles and age groups. The multivariate Cox proportional hazards regression models were conducted to estimate the adjusted relative hazard ratios (HRs) and 95% CIs of the all-cause mortality in relation to handgrip strength, after adjusting the sets of selected potential covariates (socio-demographics, health behaviors, and comorbidities). The Kaplan–Meier method was used to estimate the survival curves for all-cause mortality stratified by the quartiles of handgrip strength for each gender. Sensitivity analyses were conducted using a receiver operating characteristic (ROC) curve analysis, which was used to determine the cut-off values of the handgrip strength in association with the all-cause mortality [29]. The survival time was measured as the days from the baseline survey at 2006 until death, lost to follow up, or the end of follow-up in 2014 (censoring), whichever came first. All of the analyses were two-sided at alpha = 0.05, and performed with SPSS version 20.0 (IBM, Armonk, NY, USA).

## 3. Results

The final number of participants in this study was 9393, which was 92% of the total panel of participants. Table 1 presents the baseline characteristics of the study participants stratified by gender. Out of the total study participants of the 9393 adults aged 45 or older, 55.7% were women (*n* = 5235). The mean ages at study entry were 61.0 ± 10.7 years for the total study participants, 61.1 ± 11.0 years for women, and 60.8 ± 10.3 years for men. The mean values of the handgrip strength were 19.8 ± 5.0 kg for women, 32.5 ± 7.0 kg for men, and 25.4 ± 8.7 kg for the total study sample. The proportion of people with a lower than middle school education was higher for women (72.6%) than for men (47.8%), and the ratio of people with spouses was higher for men. The majority of men and women lived in cities, and the proportion of high-income households and employment was higher in men. The men were more likely to smoke and drink now than the women, and regular exercise and breakfast appeared to be better for the men. The proportion of obese persons was similar in men and women, and the proportion of people with three or more chronic conditions (hypertension, diabetes, cardiac disease, cerebrovascular disease, cancer, or depression) was higher in women. Table 2 presents the differences in the baseline characteristics of the study population by quartiles of handgrip strength in the women, men, and total study sample. A stronger handgrip strength was found in the adults who were younger, married, living in an urban area, and with a higher socioeconomic status (i.e., higher levels of education, income, and employment). A self-report of regular exercise was related to a stronger handgrip strength. The underweight category of BMI was related to a weaker handgrip strength, and being overweight was associated with a stronger handgrip strength. The presence of any comorbidities was related to a weaker handgrip strength in both women and men. Similarly, higher numbers of comorbidities were associated with weaker levels of handgrip strength.

Over the follow-up period (0–8 years), a total of 934 adults died, including 403 women (43.1%) and 531 men (56.9%). The all-cause mortality rate was significantly higher in men, 18.7 per 1000 person–years (95% CI: 17.2–20.4), compared to women, 11.1 per 1000 person-years (95% CI: 10.1–12.2). Table 3 demonstrates the all-cause mortality rates over the average 6.9 (±2.1) years in women and men, stratified by quartiles of handgrip strength as well as age groups. Stronger levels of handgrip strength were significantly associated with lower death rates in both women and men. As anticipated, the majority (75%) of the deceased were from 65 years or older participants. There were no significant gender differences in the death rates in the 45–54 years age group. In older age groups (i.e., 55–64 and ≥65 years), however, the men had significantly higher death rates than the women. Higher mortality rates were associated with lower socioeconomic status, risky health behaviors, and the presence of comorbidities selected in this study (data not shown).

**Figure 2:**
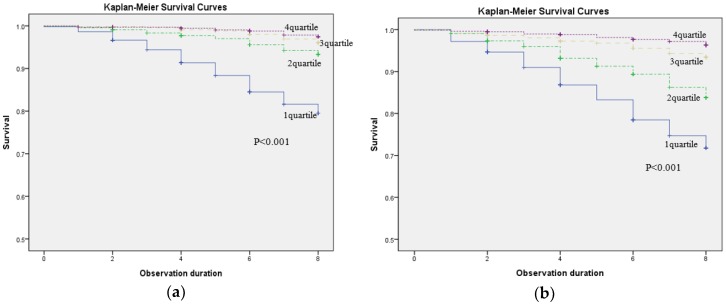
Kaplan–Meier survival curves stratified by handgrip strength quartiles: (**a**) for women and (**b**) for men.

Table 4 provides the HRs of all-cause mortality in relation to handgrip strength by women, men, and the total participants, controlling for selected covariates of age, socioeconomic factors, health behaviors, and comorbidities. Across different sets of covariates and gender, the HRs of all-cause mortality in the weakest quartile group of handgrip strength (women: <16.8 kg; and men: <28.5 kg) were consistently over three times higher than those in the strongest handgrip group (women: ≥23.0 kg; and men: ≥37.3 kg). The fully multivariate-adjusted HRs of all-cause mortality in the weakest handgrip strength group were 2.53 (95% CI: 1.67–3.84) in women, 2.62 (95% CI: 1.77–3.88) in men, and 2.81 (95% CI: 2.12–3.73) in the total participants, compared with the highest quartile group (i.e., reference group) in a respective gender group. The gender differences were suggested in the magnitude of significance in the relationship between the weaker handgrip strength and higher mortality risk: the two lowest quartiles of handgrip strength (≤32.7 kg) in men versus only the lowest quartile (<16.8 kg) in women were significantly related to mortality, compared to each gender reference group. In the total sample using the gender-specific quartiles of handgrip strength, the participants in the two lowest quartiles of handgrip strength (women ≤19.9 kg; and men ≤32.7 kg) had significantly higher HRs of all-cause mortality. We additionally ran a multivariate-adjusted Cox regression model of the all-cause mortality in relation to handgrip strength (kg) as a continuous variable, confirming the significant relationship between handgrip strength and all-cause mortality (women: HR = 0.92, 95% CI: 0.90–0.94; and men: HR = 0.94, 95% CI: 0.93–0.95) (Table S1). The Kaplan–Meier survival curves presented in Figure 2 show a better overall survival in those with a stronger handgrip strength in both women and men. The median time to event (years) from the lowest quartile (Q1) to the highest quartile (Q4) were 7.31 (95% CI: 7.21–7.40), 7.80 (95% CI: 7.75–7.85), 7.92 (95% CI: 7.89–7.95), and 7.94 (95% CI: 7.92–7.97) in women; and 7.01 (95% CI: 6.89–7.13), 7.51 (95% CI: 7.42–7.60), 7.79 (95% CI: 7.73–7.85), and 7.89 (95% CI: 7.85–7.94) in men.

**Figure 3:**
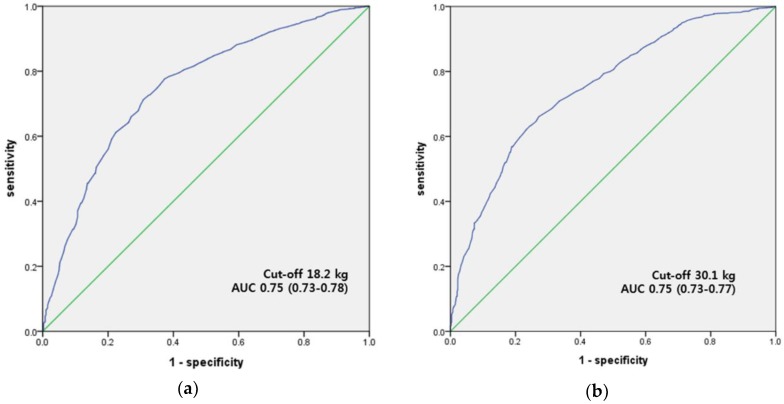
Receiver operating characteristic (ROC) curve of handgrip strength to detect death: (**a**) for women and (**b**) for men. AUC = area under the curve (95% confidence interval).

The ROC curve analyses showed a significant discriminatory accuracy in identifying the death in both women and men (women: area under the curve (AUC) = 0.75 (95% CI: 0.73–0.78), *p* < 0.001; men: AUC = 0.75 (95% CI: 0.73–0.77, *p* < 0.001). The handgrip strength values at these points were 18.2 kg and 30.1 kg in women and men, respectively (Figure 3).

## 4. Discussion

In this prospective survey study using nationwide population-based data, a weaker handgrip strength at study entry (i.e., baseline) was significantly associated with a higher all-cause mortality over the maximum eight years of follow-up in women and men aged 45 years or older in Korea. This relationship remained to be significant in various models adjusting for selected covariates, including socioeconomic factors (age, income, employment, etc.), health behaviors (smoking, exercise, BMI, etc.), and comorbidities (hypertension, cardiovascular disease, depression, etc.). Compared with women, the magnitude of associations between handgrip strength and mortality was stronger in men with a higher mortality risk.

Previous studies have shown a significant inverse relationship between handgrip strength and mortality risk in various populations of older adults largely from Western countries [6,7,30]. Those relationships were largely independent of various covariates, such as muscle mass, inflammatory markers, and comorbidity [30]. Similarly, the robust relationships found in our study were independent of age, socioeconomic factors, and self-reported health behaviors and comorbidities. While other studies focused on the elderly, including the oldest population (80 years or older), our study participants were younger, with the mean age of 61 years (range 45–98 years old). Collectively, these findings indicate the predictive value of handgrip strength for future all-cause mortality among middle-aged and older adults [31].

We observed gender differences in the relationship between handgrip strength and all-cause mortality in our study population. Most of all, the mean values of handgrip strength were significantly different between women and men (women: 19.8 kg vs. men: 32.5 kg), as expected. The men also had higher death rates across all of the quartile levels compared to those in women, showing significantly different death rates per 1000 person–years (95% CI) between women and men: 11.1 (10.1–12.2) vs. 18.7 (17.2–20.4). Accordingly, the men in the study showed the stronger relationship between handgrip strength and mortality than the women. Only a few studies have examined the gender-specific relationship with mixed findings [32,33,34]. Al Snih et al. [35], examining Mexican Americans ≥65 years of age, reported a stronger association between handgrip strength (by quartiles) and mortality after five years in the men than the women. On the other hand, Arvandi et al. [3] reported a statistically insignificant association of handgrip strength by tertiles, with an all-cause mortality among older adults over three years. The women notably showed over two times higher HRs of mortality than those in men. While the data collection period of 2008–2009 was similar to our study, the study sample was older, with a mean age of 76 years, and the sample size was smaller, with a relatively short follow-up period [3]. Our study using population-based data adds new information to the current literature on the handgrip strength–mortality relationship in the Korean population.

Little is known about the level of handgrip strength required to protect against the risk of premature death [3]. In our study, according to the quartile classification, maintaining a handgrip strength of ≥16.8 kg for women and ≥32.8 kg for men may significantly reduce the risk of premature death in Korean adults. However, this was different from the cutoff value according to the ROC curve (≥18.2 kg for women and ≥30.1 kg for men). There is still no consensus on the norm values for Korean adults with few studies. Yoo et al. [23] reported 16.4 kg for women and 28.6 kg for men as the cut-off values of weak handgrip strength, in order to determine sarcopenia among Korean population. The most recent reports with the Korean population also reported similar cut-off values o 16.8 kg for women and 28.9 kg for men [8]. The values in the weakest quartile groups (<16.8 kg in women and <28.5 kg in men) in our study are similar to the cut-off values reported by Kim et al. [9] and Yoo et al. [23]. However, further study is needed, because the cutoff value is different according to the grouping of the handgrip strength.

The handgrip strength test is commonly used to evaluate the integrated performance of the muscles by determining the maximal grip force that can be produced in one muscular contraction, further serving as a marker for general muscle strength [8]. Compared to measuring muscle mass that is relatively expensive and complex (e.g., MRI or CT), muscle strength and function simply measured by handgrip strength, as part of the defining criteria of sarcopenia, has shown its predictability to various adverse health outcomes, including morbidity and mortality [36,37]. Also, it is feasible, cheap, and acceptable to train staff to routinely measure handgrip strength [38].

Handgrip strength, differed by gender, peaks at age 35–40 years, and decreases thereafter with an accelerated decline after 60 years of age [1,7,15]. In addition to gender and age, handgrip strength is known to be affected by multiple factors, including socioeconomic status, health behaviors, and the presence of comorbidities [16,39]. In our study, these factors showed cross-sectional correlations with handgrip strength and served as covariates because of their significant relationships with mortality during the study follow-up. The selection of covariates varied across studies, largely with cross-sectional design. Consistent with previous studies [13,17], the presence of comorbidities or of having more comorbidities from self-reports were associated with a weaker handgrip strength and higher mortality, indicating the significance of incorporating muscle strengthening interventions to ongoing chronic disease management strategies. Further rigorous studies examining the longitudinal changes and relationships in these factors, handgrip strength, and mortality are warranted in order to obtain the information necessary for developing personalized prevention and care modalities.

Increasing the prevalence of sarcopenia is an important public health concern for older adults, and handgrip strength is an important component of sarcopenia. In the updated definition of sarcopenia, EWGSOP 2 emphasizes low handgrip strength as a primary indicator of probable sarcopenia [20]. Although the mechanisms underlying this association remain unknown, growing evidence suggests that simply assessing and monitoring handgrip strength during adulthood demonstrates great potentials for the public health of aging populations. It is critical to educate the middle-aged and the elderly on self-monitoring their handgrip strength and its relevant long-term health impact as early as possible, which may motivate them from early on to engage in personalized resistant exercise programs and lifestyle modification, such as nutrition and physical activity. For community-based primary practice, the handgrip strength test may be a practical screening tool for the early identification of vulnerable adults.

The limitations of this study include self-reported data, particularly on health behaviors (e.g., BMI) and comorbidities. However, these data were obtained from face-to-face interviews by trained interviewers, which is likely to improve the validity of the data over self-administered surveys. Because of the nature of nationwide, population-based survey study, the measurements for the data were rather simple and crude. The relationships of the baseline variables with handgrip strength and mortality shown in this study were consistent with previous studies that used other standardized instruments [3,6,18,31]. Also, we were not able to address other covariates, such as the severity and chronicity of diseases. In addition, we were not able to examine the associations among handgrip strength and cause-specific mortality outcomes, because of the lack of data on causes of death in this survey dataset. The strengths of this study include using a prospective study design with a relatively longer follow-up, and population-based interview data with great participation rates (>90%) over the study follow-up. To the best of our knowledge, this is the first study to demonstrate the longitudinal and independent relationship of handgrip strength with all-cause mortality in middle-aged and old Korean adults, controlling for various covariates.

## 5. Conclusions

A weaker handgrip strength was significantly associated with a higher risk of all-cause mortality in Korean adults in both women and men. The significant relationships were not influenced by any effects of age, socioeconomic status, and selected health behaviors and comorbidities. The relationships between handgrip strength and death were stronger in men compared with those in women, indicating potential differences by gender. Along with other Korean studies, this study suggests that maintaining a handgrip strength of at least 16.8 kg for women and 32.8 kg for men may be critical for the healthy aging and longevity of the Korean population. The findings are particularly encouraging, in that simply assessing and monitoring handgrip strength may be a promising tool for identifying subgroups of the vulnerable and at-risk populations. Further research using rigorous methodology, including the longitudinal assessment of handgrip strength, is warranted in order to develop tailored interventions for improving and maintaining the muscle strength of Korean adults.

## Figures and Tables

**Table 1:** Baseline characteristics of the study population.

	Women (*n* = 5235)	Men (*n* = 4158)	Total (*n* = 9393)
Handgrip strength, kg,	19.8 ± 5.0 (19.6–19.9)	32.5 ± 7.0 (32.3–32.7)	25.4 ± 8.7 (25.2–25.6)
Mean ± SD (95% CI)			
Age, years,	61.1 ± 11.0 (60.8–61.4)	60.8 ± 10.3 (60.4–61.1)	61.0 ± 10.7 (60.7–61.2)
Mean ± SD (95% CI)			
Age groups (%, 95% CI)			
45–54 years	1797 (34.3, 37.2–39.8)	1396 (33.6, 32.1–35.0)	3193 (34.0, 33.0–34.9)
55–64 years	1424 (27.2, 26.0–28.4)	1198 (28.8, 27.4–30.1)	2622 (27.9, 27.0–28.8)
≥65 years	2014 (38.5, 33.1–35.6)	1564 (37.6, 36.0–39.1)	3578 (38.1, 37.1–39.0)
Education (%, 95% CI)			
≤Middle school	3799 (72.6, 71.4–73.8)	1986 (47.8, 46.2–49.3)	5785 (61.6, 60.6–62.6)
High school	1168 (22.3, 21.2–23.4)	1432 (34.4, 33.0–36.1)	2600 (27.7, 26.8–28.6)
≥College	268 (5.1, 4.5–5.7)	740 (17.8, 16.7–19.0)	1008 (10.7, 10.1–11.4)
Marital status (%, 95% CI)			
Married	3607 (68.9, 67.7–70.2)	3827 (92.0, 91.2–92.9)	7434 (79.1, 78.4–79.9)
Divorced/widowed/never married	1628 (31.1, 29.2–31.7)	331 (8.0, 6.9–9.0)	1959 (20.9, 19.8–21.8)
Residence (%, 95% CI)			
Urban	4100 (78.3, 77.2–79.5)	3227 (77.6, 76.3–78.9)	7327 (78.0, 77.1–78.9)
Rural	1135 (21.7, 20.5–22.8)	931 (22.4, 21.1–23.7)	2066 (22.0, 21.1–22.9)
Income (%, 95% CI)			
Low	1445 (27.6, 26.3–28.8)	946 (22.8, 21.4–24.0)	2391 (25.5, 24.6–26.3)
Middle	2572 (49.1, 47.8–50.5)	2050 (49.3, 47.7–50.9)	4622 (49.2, 48.2–50.2)
High	1218 (23.3, 22.2–24.4)	1162 (27.9, 26.6–29.4)	2380 (25.3, 24.5–26.2)
Employment (%, 95% CI)	1341 (25.6, 24.3–26.8)	2478 (59.6, 58.1–61.0)	3819 (40.7, 39.6–41.6)
Smoking (%, 95% CI)			
Non-smoker	5038 (96.2, 95.7–96.8)	1616 (38.9, 37.4–40.3)	6654 (70.8, 69.9–71.8)
Former smoker	27 (0.5, 0.3–0.7)	859 (20.7, 19.5–21.9)	886 (9.4, 8.9–10.0)
Current smoker	170 (3.2, 2.8–3.8)	1683 (40.5, 39.0–41.9)	1853 (19.7, 18.9–20.5)
Drinking (%, 95% CI)			
Non-drinker	4105 (78.4, 77.7–79.5)	994 (23.9, 22.6–25.4)	5099 (54.3, 53.2–55.3)
Former drinker	116 (2.2, 1.8–2.6)	470 (11.3, 10.4–12.3)	586 (6.2, 5.8–6.7)
Current drinker	1014 (19.4, 18.3–20.4)	2694 (64.8, 63.2–66.2)	3708 (39.5–38.5–40.5)
Regular exercise (%, 95% CI)	1925(36.8, 35.4–38.1)	1827 (43.9, 42.5–45.5)	3752 (39.9, 39.0–41.0)
Eating breakfast (%, 95% CI)	4880 (93.2, 92.6–93.9)	3956 (95.1, 94.5–95.8)	8836 (94.1, 93.6–94.5)
Body mass index (BMI), kg/m^2^,			
Mean ± SD (95% CI)	23.3 ± 3.5 (23.2–23.4)	23.8 ± 10.7 (23.5–24.2)	23.6 ± 7.6 (23.4–23.7)
BMI group (%, 95% CI)			
<18.5	142 (2.7, 2.3–3.2)	59 (1.4, 1.1–1.8)	201 (2.1, 1.8–2.4)
18.5–24.9	1192 (22.8, 21.7–23.9)	876 (21.1, 19.8–22.3)	2068 (22.0, 21.2–22.8)
25–29.9	3705 (70.8, 69.5–72.0)	3088 (74.3, 72.9–75.7)	6793 (72.3, 71.4–73.3)
≥30	196 (3.7, 3.2–4.3)	135 (3.2, 2.7–3.8)	331 (3.5, 3.2–3.9)
Hypertension (%, 95% CI)	1526 (29.1, 27.9–30.3)	1008 (24.2, 23.0–25.5)	2534 (27.0, 26.1–27.8)
Diabetes (%, 95% CI)	588 (11.2, 10.4–12.1)	501 (12.0, 11.1–13.0)	1089 (11.6, 10.9–12.2)
Cardiac disease (%, 95% CI)	266 (5.1, 4.5–5.7)	192 (4.6, 4.0–5.3)	458 (4.9, 4.5–5.3)
Cerebrovascular disease	100 (1.9, 1.5–2.3)	124 (3.0, 2.5–3.5)	224 (2.4, 2.1–2.7)
(%, 95% CI)			
Cancer (%, 95% CI)	121 (2.3, 1.9–2.7)	83 (2.0, 1.6–2.4)	204 (2.2, 1.9–2.5)
Depression (%, 95% CI)	716 (13.7, 12.8–14.7)	295 (7.1, 6.3–7.9)	1011 (10.8, 10.2–11.4)
# of comorbidities (%, 95% CI)			
0	2903 (55.5, 54.2–56.8)	2553 (61.4, 59.9–62.9)	5456 (58.1, 57.1–59.1)
1	1572 (30.0, 28.8–31.3)	1124 (27.0, 25.6–28.4)	2696 (28.7, 27.8–29.6)
2	560 (10.7, 9.8–11.5)	381 (9.2, 8.3–10.1)	941 (10.0, 9.4–10.6)
3 or more	200 (3.8, 3.3–4.3)	100 (2.4, 2.0–2.9)	300 (3.2, 2.8–3.6)

Notes: CI = confidence interval; SD = standard deviation.

**Table 2:** Differences in baseline characteristics of the study population by quartiles of handgrip strength.

	Handgrip Strength														
	Women (*n* = 5235, 55.7%)					Men (*n* = 4158, 44.3%)					Total (*n* = 9393, 100%)				
	1Q_ Lowest (*n* = 1289)	2Q_ Low-Middle (*n* = 1263)	3Q_ High-Middle (*n* = 1308)	4Q_ Highest (*n* = 1375)	*p*	1Q_ Lowest (*n* = 1031)	2Q_ Low-Middle (*n* = 1031)	3Q_ High-Middle (*n* = 1023)	4Q_ Highest (*n* = 1073)	*p*	1Q_ Lowest (*n* = 2320)	2Q_ Low-Middle (*n* = 2294)	3Q_ High-Middle (*n* = 2331)	4Q_ Highest (*n* = 2448)	*p*
Age, years,	70.0 ± 10.4	62.9 ± 9.7	58.0 ± 9.1	54.0 ± 7.9	<0.001	69.0 ± 9.2	62.9 ± 9.3	57.9 ± 8.6	53.6 ± 7.0	<0.001	69.6 ± 9.9	62.9 ± 9.5	57.9 ± 8.9	53.8 ± 7.5	<0.001
Mean ± SD															
Age groups (%)					<0.001					<0.001					<0.001
45–54 years	121 (9.4)	300 (23.8)	536 (41.0)	840 (61.1)		89 (8.6)	213 (20.7)	422 (41.3)	672 (62.6)		210 (9.1)	513 (22.4)	958 (41.1)	1512 (61.8)	
55–64 years	234 (18.2)	372 (29.5)	450 (34.4)	368 (26.8)		204 (19.8)	338 (32.8)	348 (34.0)	308 (28.7)		438 (18.9)	710 (31.0)	798 (34.2)	676 (27.6)	
≥ 65 years	934 (72.5)	591 (46.8)	322 (24.6)	167 (12.1)		738 (71.6)	480 (46.6)	253 (24.7)	93 (8.7)		1672 (72.1)	1071 (46.7)	575 (24.7)	260 (10.6)	
Education (%)					<0.001					<0.001					<0.001
≤Middle school	1160 (90.0)	1,007 (79.7)	887 (67.8)	745 (54.2)		723 (70.1)	552 (53.5)	400 (39.1)	311 (29.0)		1883 (81.2)	1559 (68.0)	1287 (55.2)	1056 (43.1)	
High school	108 (8.4)	214 (16.9)	334 (25.5)	512 (37.2)		218 (21.1)	323 (31.3)	394 (38.5)	497 (46.3)		326 (14.1)	537 (23.4)	728 (31.2)	1009 (41.2)	
≥College	21 (1.6)	42 (3.3)	87 (6.7)	118 (8.6)		90 (8.7)	156 (15.1)	229 (22.4)	265 (24.7)		111 (4.8)	198 (8.6)	316 (13.6)	383 (15.6)	
Marital status (%)					<0.001					<0.001					<0.001
Married	596 (46.2)	829 (65.6)	1014 (77.5)	1168 (84.9)		909 (88.2)	944 (91.6)	963 (94.1)	1011 (94.2)		1505 (64.9)	1773 (77.3)	1977 (84.8)	2179 (89.0)	
Divorced/widowed/	693 (53.8)	434 (34.4)	294 (22.5)	207 (15.1)		122 (11.8)	87 (8.4)	60 (5.9)	62 (5.8)		815 (35.1)	521 (22.7)	354 (15.2)	269 (11.0)	
never married															
Residence					0.002					<0.001					<0.001
Urban	972 (75.4)	976 (77.3)	1035 (79.1)	1117 (81.2)		733 (71.1)	783 (75.9)	815 (79.7)	896 (83.5)		1705 (73.5)	1759 (76.7)	1850 (79.4)	2013 (82.2)	
Rural	317 (24.6)	287 (22.7)	273 (20.9)	258 (18.8)		298 (28.9)	248 (24.1)	208 (20.3)	177 (16.5)		615 (26.5)	535 (23.3)	481 (20.6)	435 (17.8)	
Income					<0.001					<0.001					<0.001
Low	491 (38.1)	386 (30.6)	308 (23.5)	260 (18.9)		374 (36.3)	250 (24.2)	173 (16.9)	149 (13.9)		865 (37.3)	636 (27.7)	481 (20.6)	409 (16.7)	
Middle	616 (47.8)	653 (51.7)	659 (50.4)	644 (46.8)		534 (51.8)	528 (51.2)	515 (50.3)	473 (44.1)		1150 (49.6)	1181 (51.5)	1174 (50.4)	1117 (45.6)	
High	182 (14.1)	224 (17.7)	341 (26.1)	471 (34.3)		123 (11.9)	253 (24.5)	335 (32.7)	451 (42.0)		305 (13.1)	477 (20.8)	676 (29.00	922 (37.7)	
Employment (%)	163 (12.6)	288 (22.8)	390 (29.8)	500 (36.4)	<0.001	338 (32.8)	557 (54.0)	702 (68.6)	881 (82.1)	<0.001	501 (21.6)	845 (36.8)	1092 (46.8)	1381 (56.4)	<0.001
Smoking (%)					0.188					0.001					0.001
Non-smoker	1230 (95.4)	1208 (95.6)	1262 (96.5)	1338 (97.3)		389 (37.7)	398 (38.6)	411 (40.2)	418 (39.0)		1619 (69.8)	1606 (70.0)	1673 (71.8)	1756 (71.7)	
Former smoker	8 (0.6)	9 (0.7)	6 (0.5)	4 (0.3)		256 (24.8)	224 (21.7)	190 (18.6)	189 (17.6)		264 (11.4)	233 (10.2)	196 (8.4)	193 (7.9)	
Current smoker	51 (4.0)	46 (3.6)	40 (3.1)	33 (2.4)		386 (37.4)	409 (39.7)	422 (41.3)	466 (43.4)		437 (18.8)	455 (19.8)	462 (19.8)	499 (20.4)	
Drinking (%)					<0.001					<0.001					<0.001
Non-drinker	1084 (84.1)	994 (78.7)	1011 (77.3)	1016 (73.9)		274 (26.6)	260 (25.2)	231 (22.6)	229 (21.3)		1358 (58.5)	1254 (54.7)	1242 (53.3)	1245 (50.9)	
Former drinker	42 (3.3)	35 (2.8)	21 (1.6)	18 (1.3)		205 (19.9)	122 (11.8)	84 (8.2)	59 (5.5)		247 (10.6)	157 (6.8)	105 (4.5)	77 (3.1)	
Current drinker	163 (12.6)	234 (18.5)	276 (21.1)	341 (24.8)		552 (53.5)	649 (62.9)	708 (69.2)	785 (73.2)		715 (30.8)	883 (38.5)	987 (42.2)	1126 (46.0)	
Regular exercise (%)	347 (26.9)	437 (34.6)	542 (41.4)	599 (43.6)	<0.001	347 (33.7)	459 (44.5)	479 (46.8)	542 (50.5)	<0.001	649 (29.9)	896 (39.1)	1021 (43.8)	1141 (46.6)	<0.001
Eating breakfast (%)	1225 (95.0)	1188 (94.1)	1205 (92.1)	1262 (91.8)	0.002	988 (95.8)	987 (95.7)	976 (95.4)	1005 (93.7)	0.070	2213 (95.4)	2175 (94.8)	2181 (93.6)	2267 (92.6)	<0.001
Body mass index (BMI), kg/m^2^,					<0.001					<0.001					<0.001
Mean ± SD	22.8 ± 3.2	23.3 ± 3.4	23.5 ± 3.9	23.8 ± 3.4		22.8 ± 11.1	23.5 ± 10.0	24.1 ± 8.1	24.9 ± 12.9		22.8 ± 7.7	23.4 ± 7.2	23.7 ± 6.1	24.3 ± 8.9	
BMI group (%)					<0.001					<0.001					<0.001
<18.5	33 (2.6)	39 (3.1)	26 (2.0)	44 (3.2)		14 (1.4)	11 (1.1)	14 (1.4)	20 (1.9)		47 (2.0)	50 (2.2)	40 (1.7)	64 (2.6)	
18.5–24.9	255 (19.8)	274 (21.7)	316 (24.2)	347 (25.2)		125 (12.1)	186 (18.0)	250 (24.4)	315 (29.4)		380 (16.4)	460 (20.1)	566 (24.3)	662 (27.0)	
25–29.9	902 (70.0)	902 (71.4)	937 (71.6)	964 (70.1)		816 (79.1)	798 (77.4)	744 (72.7)	730 (68.0)		1718 (74.1)	1700 (74.1)	1681 (72.1)	1694 (69.2)	
≥30	99 (7.7)	48 (3.8)	29 (2.2)	20 (1.5)		76 (7.4)	36 (3.5)	15 (1.5)	8 (0.7)		175 (7.5)	84 (3.7)	44 (1.9)	28 (1.1)	
Hypertension (%)	536 (41.6)	396 (31.4)	341 (26.1)	253 (18.4)	<0.001	331 (32.1)	281 (27.3)	212 (20.7)	184 (17.1)	<0.001	867 (37.4)	667 (29.5)	553 (23.7)	437 (17.9)	<0.001
Diabetes (%)	230 (17.8)	164 (13.0)	117 (8.9)	77 (5.6)	<0.001	179 (17.4)	137 (13.3)	101 (9.9)	84 (7.8)	<0.001	409 (17.6)	301 (13.1)	218 (9.4)	161 (6.6)	<0.001
Cardiac disease (%)	117 (9.1)	76 (6.0)	45 (3.4)	28 (2.0)	<0.001	76 (7.4)	55 (5.3)	33 (3.2)	28 (2.6)	<0.001	193 (8.3)	131 (5.7)	78 (3.3)	56 (2.3)	<0.001
Cerebrovascular disease (%)	42 (3.3)	20 (1.6)	29 (2.2)	9 (0.7)	<0.001	64 (6.2)	39 (3.8)	12 (1.2)	9 (0.8)	<0.001	106 (4.6)	59 (2.6)	41 (1.8)	18 (0.7)	<0.001
Cancer (%)	39 (3.0)	30 (2.4)	25 (1.9)	27 (2.0)	0.204	33 (3.2)	18 (1.7)	24 (2.3)	8 (0.7)	0.001	72 (3.1)	48 (2.1)	49 (2.1)	35 (1.4)	0.001
Depression (%)	275 (21.3)	175 (13.9)	163 (12.5)	103 (7.5)	<0.001	118 (11.4)	75 (7.3)	48 (4.7)	54 (5.0)	<0.001	393 (16.9)	250 (10.9)	211 (9.1)	157 (6.4)	<0.001
# of comorbidities (%)					<0.001					<0.001					<0.001
0	491 (38.1)	661 (52.3)	781(59.7)	970 (70.5)		496 (48.1)	595 (57.7)	682 (66.7)	790 (72.7)		987 (42.5)	1256 (54.8)	1463 (62.8)	1750 (71.5)	
1	465 (36.1)	408 (32.3)	372(28.4)	327 (23.8)		329 (31.9)	304 (29.5)	266 (26.0)	225 (21.0)		794 (34.2)	712 (31.0)	638 (27.4)	552 (22.5)	
2	239 (18.5)	135 (10.7)	119(9.1)	67 (4.9)		154 (14.9)	103 (10.0)	62 (6.1)	62 (5.8)		393 (16.9)	238 (10.4)	181 (7.8)	129 (5.3)	
3 or more	94 (7.3)	59 (4.7)	36(2.8)	11 (0.8)		52 (5.0)	29 (2.8)	13 (1.3)	6 (0.6)		146 (6.3)	88 (3.8)	49 (2.1)	17 (0.7)	

Notes: Q = quartile; SD = standard deviation.

**Table 3:** All-cause mortality in women and men by quartiles of handgrip strength and age groups.

	Women (*n* = 5235)			Men (*n* = 4158)			Total (*n* = 9393)
	Total *n*	Death *n* (%)	Death Rate per 1000 p-y (95% CI)	Total *n*	Death *n* (%)	Death Rate per 1000 p–y (95% CI)	Death Rate per 1000 p–y (95% CI)
Handgrip Strength							
Q1 (lowest)	1289	247 (19.2)	29.1 (25.7–33.0)	1031	281 (27.3)	42.0 (37.4–47.2)	34.8 (32.0–37.9)
Q2	1263	81 (6.4)	9.1 (7.3–11.3)	1031	153 (14.8)	21.7 (18.5–25.4)	14.7 (12.9–16.7)
Q3	1308	45 (3.4)	4.9 (3.7–6.6)	1023	62 (6.1)	8.6 (6.7–11.0)	6.5 (5.4–7.9)
Q4 (highest)	1375	30 (2.2)	3.1 (2.2–4.4)	1073	35 (3.3)	4.7 (3.4–6.5)	3.8 (3.0–4.8)
Age							
45–54 years	1797	24 (1.3)	1.9 (1.3–2.8)	1396	43 (3.1)	4.5 (3.3–6.1)	3.0 (2.4–3.8)
55–64 years	1424	48 (3.4)	4.7 (3.5–6.2)	1198	100 (8.3)	11.8 (9.7–14.4)	7.9 (6.7–9.3)
≥65 years	2014	331 (16.4)	24.3 (21.8–27.1)	1564	388 (24.8)	37.4 (33.9–41.3)	30.0 (27.9–32.3)
Total	5235	403 (43.1)	11.1 (10.1–12.2)	4158	531 (56.9)	18.7 (17.2–20.4)	14.4 (13.5–15.4)

Notes: p–y = person–years; CI = confidence interval; Q = quartile.

**Table 4:** Hazard ratios of all-cause mortality in relation to handgrip strength over eight-year study follow-up.

	Handgrip Strength			
	Q 1 (95% CI) (Lowest)	Q2 (95% CI)	Q3 (95% CI)	Q4 (95% CI) (Highest)
Women	2.53 (1.67–3.84)	1.22 (0.79–1.89)	1.02 (0.64–1.63)	1.00 (Reference)
Men	2.62 (1.77–3.88)	2.02 (1.37–2.98)	1.16 (0.76–1.77)	1.00 (Reference)
Total	2.81 (2.12–3.73)	1.72 (1.29–2.30)	1.13 (0.83–1.55)	1.00 (Reference)

Models were adjusted for socioeconomic factors, health behaviors, and comorbidities; Notes: Q = quartile; CI = confidence interval.

## Supplementary Materials

The following are available online at https://www.mdpi.com/1660-4601/16/5/740/s1, Table S1. Handgrip strength and hazard ratio of all-cause mortality over 8-year study follow-up (as continuous variable).
