# Supplementary material for: Handgrip Strength and All-Cause Mortality in Middle-Aged and Older Koreans
Source: Int J Environ Res Public Health. 2019 Mar 1;16(5):740. doi: 10.3390/ijerph16050740 (PMC6427792; doi:10.3390/ijerph16050740)
Supplement: Supplementary file 1 [file ijerph-16-00740-s001.pdf]

Supplementary Material

Table S1. Handgrip strength and hazard ratio of all-cause mortality over 8-year study follow-up (as continuous variable)

	Handgrip strength (kg)			
	**B**	**SD**	**HR**	**95% CI**
**Women**	-0.083	0.011	0.92	0.90-0.94
**Men**	-0.061	0.007	0.94	0.93-0.95
**Total**	-0.024	0.006	0.98	0.97-0.99

Model is adjusted for age, socioeconomic factors, health behaviors, and comorbidities
CI = confidence interval
